# Assessment of the Bacterial Diversity of Aircraft Water: Identification of the Frequent Fliers

**DOI:** 10.1371/journal.pone.0170567

**Published:** 2017-01-23

**Authors:** Harald Handschuh, Michael P. Ryan, Jean O’Dwyer, Catherine C. Adley

**Affiliations:** 1 Microbiology Laboratory, Department of Chemical Sciences, School of Natural Sciences, University of Limerick, Limerick, Ireland; 2 Industrial Biochemistry Programme, Department of Chemical Sciences, School of Natural Sciences, University of Limerick, Limerick, Ireland; 3 Department of Biological Sciences, School of Natural Sciences, University of Limerick, Limerick, Ireland; Fudan University, CHINA

## Abstract

The aim of this study was to determine and identify bacteria inhabiting the supply chain of an airline’s drinking water using phenotypic and 16S rDNA sequence-based analysis. Water samples (*n* = 184) were sourced from long-haul and short-haul aircraft, the airline water source and a water service vehicle. In total, 308 isolates were characterised and their identity determined, which produced 82 identified bacterial species belonging to eight classes: γ-Proteobacteria; β-Proteobacteria; α-Proteobacteria; Bacilli; Actinobacteria; Flavobacteria; Sphingobacteria and Cytophaga. Statistical differences in bacterial diversity were found to exist across sampling locations (X^2^ = 39.220, p = 0.009) and furthermore, differences were observed (X^2^ = 15.475, p = 0.030) across aircraft type (long- or short-haul). This study demonstrates the diverse nature of microorganisms within the aircraft drinking water supply chain. To the best of our knowledge, this is the most extensive study undertaken to date of microbial diversity in aircraft drinking water.

## Introduction

Water can support and host an array of pathogenic [1] and non-pathogenic microorganisms, amongst which are bacteria, fungi, viruses, protozoa, helminths and Schistosoma [2]. As such, a fundamental aspect of safeguarding public health is the routine microbiological analysis of drinking water for early detection of microbial contamination and assessing sources of deterioration in quality. Bacteria found in fresh water, including naturally occurring aquatic bacteria, can come from many different habitats, or from soil, human or animal intestinal tracts; the primary taxonomic groups are α-Proteobacteria, β-Proteobacteria, Bacteroidetes [3, 4] and γ-Proteobacteria [5]. Aquatic bacteria are predominantly Gram-negative and are comprised largely of genera such as *Pseudomonas*, *Acinetobacter Flavobacterium*, and *Cytophaga*, along with some types of Gram-positive bacteria such as coryneform bacteria, *Micrococcus* and *Bacillus* [6]. Pyrosequencing of drinking water from a US university campus to analyse its microbial ecology revealed that α-Proteobacteria and β-Proteobacteria dominated this bacterial community [7].

Bacteria can exploit very dilute solutions of organic matter [8], and can form microbial aggregates in the water system that are capable of sheltering bacteria from contact with chlorine disinfectants, including systems with a controlled constant residual chlorine level [9, 10]. The nature of bacterial communities in water has reportedly changed following chlorine disinfection, with evidence of decreased quantities of α-Proteobacteria, β-Proteobacteria and γ-Proteobacteria [11], and increased levels of β-Proteobacteria during winter months [12]. Microorganisms can continue to be present in drinking water even post-treatment; they may be incapacitated by the treatment process, but may recover from this and grow in water distribution system biofilms [13]. Sections of biofilm can subsequently break off, in a process known as sloughing, and enter the main body of water [14].

A fundamental aspect of safeguarding public health is the routine microbiological analysis of drinking water and early detection of microbial contamination. This requirement is enforced in Europe through the European Union (EU) quality of water legislation and food safety regulations [15, 16], and likewise, in the United States of America, through the Environmental Protection Agency (US-EPA), Safe Drinking Water Act and the Drinking Water Rule For Aircraft [17, 18]. International recommendations for risk management have been formulated by the World Health Organisation (WHO) for hazards compromising drinking water [19, 20].

Drinking water supply at airports generally originates from a ground or surface water source, which is treated and filtered at regional state-owned and operated water treatment plants, or by private water providers under the auspices of health authorities and EPAs. Some airports have water reservoirs, supplied with potable water from the municipal water system to make substantial amounts of water available for large airport infrastructures and their reticulations, but also in case of emergencies. Chlorine is boosted prior to water distribution form the reservoir to ensure recommended residual levels are maintained. Drinking water is then either supplied and stored in intermediate storage tanks, or distributed directly throughout the airport to terminals and other airport facilities, including water collection or fill points. Drinking water for aircraft is then collected from those fill points by a Water Service Vehicle (WSV) with a capacity of between several hundred and several thousand litres of water. Once filled, a WSV is driven to an aircraft in order to upload the water into the aircraft potable water tank by means of a stainless steel pump operated on the WSV. All passenger aircraft are equipped with one or more potable water tanks, which act as reservoirs for the on-board water distribution system, and are usually topped-up rather than being filled from empty. Each tank is constructed from a light, but very robust material to reduce weight, whilst retaining the ability to withstand pressure and rapid temperature changes encountered during normal flight operations. From the tank, drinking water is then distributed on board, predominantly via stainless steel piping, to lavatory and galley outlet points, i.e. taps and water heater, via a carbon filter in each galley. The filters are contained within stainless steel filter housing, and their function is to remove unwanted taste and chlorine odour from the water, whilst filtering out impurities and reducing limescale deposits in water heaters.

The water supply on board aircraft is used by passengers in multiple ways, for example, consumed as a component of hot beverages and soups, for reconstituting baby food or imbibed simply as a glass of water for the intake of medicine. The same water is also used by passengers via the medium of the hot towels used for refreshing purposes by applying them to the face and hands; likewise, the water is utilised in rinsing food equipment, for washing hands, brushing teeth and flushing lavatories. New facilities in first class travel include the availability of showers.

Outbreaks of water-borne diseases during travel on cruise ships have been reported during 1970 and 2003, with the pathogen enterotoxigenic *Escherichia coli* (ETEC) most frequently identified as being the causative agent, along with *Salmonella* spp., *Shigella* spp., *Cryptosporidium* spp., and *Giardia intestinalis* and Norovirus [21]. However, no documented evidence could be found to date that would indicate conclusively that passengers have been infected by consuming contaminated drinking water on board aircraft [22]. Furthermore, no cases of illness or disease have been reported thus far as being the result of contaminated water on US carriers, according to a statement made in 2011 by the US Air Transport Association, now known as Airlines for America [23]. However, it is conceivable that there are multiple reasons why the transmission of infectious diseases is probably less frequently reported, a most likely one being that most disease incubation periods are longer than the duration of the respective flights [24].

This research is a continuation of work by Handschuh *et al*. (2015) [25], whereby the microbial quality of water on board two aircraft were systematically analysed, including the water originating from the WSV and the source water. Results demonstrated that water on long-haul flights was significantly poorer in terms of microbial quality than on short-haul flights (p = 0.015), and further analysis indicated that the water service vehicle was a significant source of increased microbial load in aircraft. General bacterial diversity was also assessed for both aircraft, and the multiplicity of the bacterial communities prompted this current research. The aim of this paper was, therefore, to assess the bacterial diversity within an airline fleet (39 aircraft) and, through classification and subsequent statistical analysis, to provide an overview of both the diversity of the microbial ecology in this unique environment, thereby highlighting areas of heterogeneous diversity, potentially requiring remediate action.

## Materials and Methods

### Water Sampling

Individual water samples (*n* = 184) were taken during the period from January 2009 to June 2013 (Table 1). Water sampling was undertaken using the standard International Organization for Standardization (ISO) protocols for general water sampling: BS ISO 5667–21:2010 [26] and tanker sampling, BS ISO 5667–5:2006 [27]. Samples were taken from both forward and aft galleys on 31 short-haul aircraft (short-range aircraft used for European destinations) and 8 long-haul aircraft (long-range aircraft used for transatlantic destinations) across an airline fleet. This sampling was carried out every 48 days as part of the aircraft maintenance programme. Sampling was also undertaken on the water service vehicle (WSV) and the airport fill-point (water source- The airport water supply originates from a ground or surface water source which is filtered and treated at a water treatment plant operated by the relevant municipal authority. The potable water is then piped, as part of the municipal water system, to the airport water reservoir where the water is held, and chlorine levels are boosted with sodium hypochlorite (NaClO) by the relevant airline on a monthly basis. Permissions for sampling were given by the airline company managing the aircraft fleet and by the airport managing authority. All analysis was carried out in an onsite laboratory.

**Table 1:** Water Sampling Location, Number of Samples and Identifications.

Sampling Period January 2009 –June 2013		
Location	Number of Water Samples	Number of Identifications
Long-haul AC, Fwd & Aft (*n* = 8)	64	110
Short-haul AC, Fwd & Aft (*n* = 31)	92	151
Water Service Vehicle (WSV)	18	27
Water Source	10	21
Total	184	308

**Abbreviations:** Fwd—forward galley, Aft—aft galley, AC -Aircraft

All taps and hoses from which samples were taken were disinfected by means of clinical alcohol disinfectant wipes (Pal International, Leicestershire, UK), flushed through and then sampled. All airplane galley taps (flow rate of 3 L/min) were flushed for 3 mins; the WSV (flow rate of 15 L/min) and the water source (flow rate of 120 L/min) were both flushed for 30 secs. Pre-sterilised sampling bottles (Sterilin^®^, UK) were used to collect 250 ml samples from each of the sample points. Isolation of bacteria originated from water samples taken during specific water sampling activities [25] and routine aircraft water sampling.

### Microbiological Analysis

All isolates originated from heterotrophic plate count (HPC) analysis of aircraft water, WSV and water source samples. Serial dilutions were performed using Tryptone based diluent (AES Laboratoire, Bruz Cedex, France) on all water samples. Yeast Extract Agar (YEA) (Oxoid) and R2A (Oxoid, UK) plates were then inoculated using the pour plate technique and incubated at 37°C for 44 ± 4 hours and at 22°C for 68 ± 4 hours. Bacterial colonies were selected according to shape, pigmentation and size to capture the maximum diversity of bacteria in the aircraft, water service vehicle and source water samples. Isolated colonies were streaked onto YEA or R2A plates and incubated for 5 days at the same temperature as the original colony growth. They were subsequently re-streaked onto the same media to obtain pure cultures.

### Identification of Bacterial Isolates

After initial isolation, preliminary testing was carried out (Gram staining, oxidase and catalase testing). The API 20NE (a manual biochemical identification test, BioMérieux, France), was used to identify 78 isolations. Subsequently 97 isolations were identified using the automated biochemical identification test VITEK (BioMérieux, France) applying Non-Fermenter cards (NFC), the Gram-Negative Identification plus (GNI+) and the Gram-Positive Identification (GPI) test cards. All tests were carried out according to the manufacturer’s instructions. Where inconclusive results were obtained using the phenotypic methods, the MicroSeq500® (16*S* rDNA identification system that sequences the first 527 bp of the gene, Applied Biosystems^TM^) was used to identify the isolates.

### Bioinformatic Analysis

The 16*S* rDNA gene sequences (~450bp) found in this study were compared with gene sequences from the GenBank database using the Basic Local Alignment Search Tool (BLAST) [28] and the sequences were then aligned using the ClustalW programme [29]. Phylogenetic and molecular evolutionary analyses were conducted using genetic distance-based neighbour-joining algorithms [30] within MEGA version 6.1 (http://www.megasoftware.net) [31,32]. The confidence of the tree topology has been estimated through 1000 Bootstrap analysis duplications.

### Statistical Analysis

Prior to analysis, all independent variables were assessed for normality using the Kolmogorov–Smirnov test, in concurrence with Q-Q plots. All data was found to exhibit a non-normal distribution and thus non-parametric analyses were employed. Pearson’s χ2 test was used to test whether observed differences in bacterial diversity (based on bacterial classification) between sampling locations, i.e., long/short haul aircraft, water service vehicle and the water source were statistically significant. Furthermore, for assessment of diversity within the aircraft, i.e., the forward and aft galleys were also assessed for statistical difference using Pearson’s Chi Square analysis. SPSS® version 22 (IBM Corporation, New York, NY, USA) was employed for all statistical analyses and the significance level was set at 95% (p ≤ 0.05) for all analyses.

## Results and Discussion

**Fig 1:**
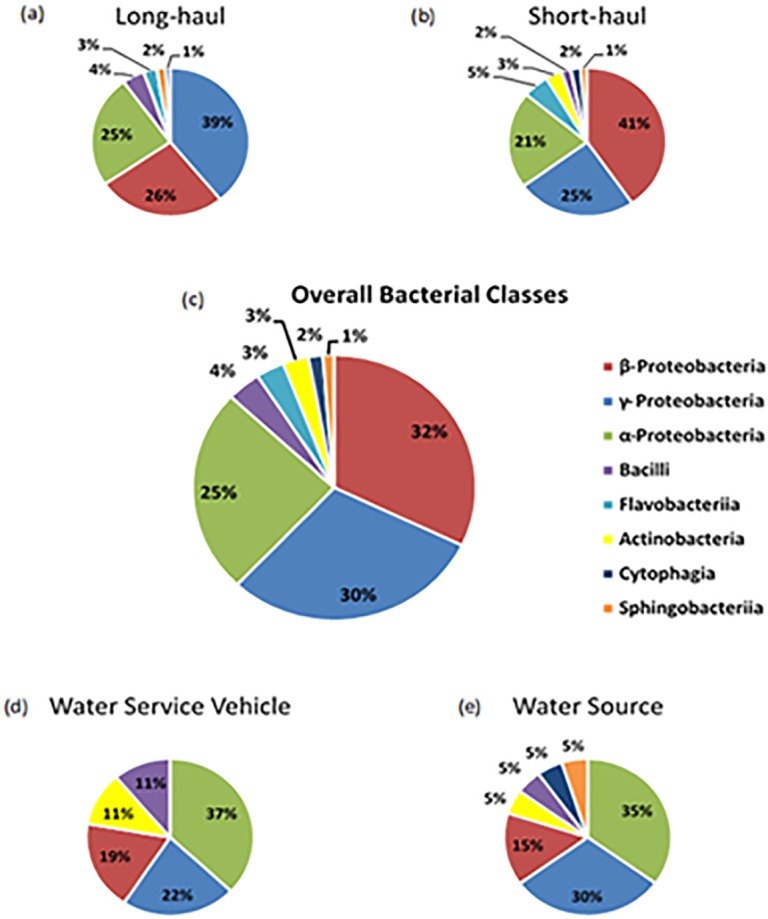
Distribution of bacterial class (% contribution) across sampling location: Long and short-haul aircraft, Water Service Vehicle (WSV) and Source water.

A total of 308 identified bacterial isolates were recovered from routine aircraft water samples collected along the water supply chain from the Water Source, Water Service Vehicle (WSV), and from water taps in the galleys of both short- and long-haul designated aircraft. Bacteria identified belonged to eight classes (Fig 1): γ-Proteobacteria; β-Proteobacteria; α-Proteobacteria; Bacilli; Actinobacteria; Flavobacteria; Sphingobacteria and Cytophaga.

**Fig 2:**
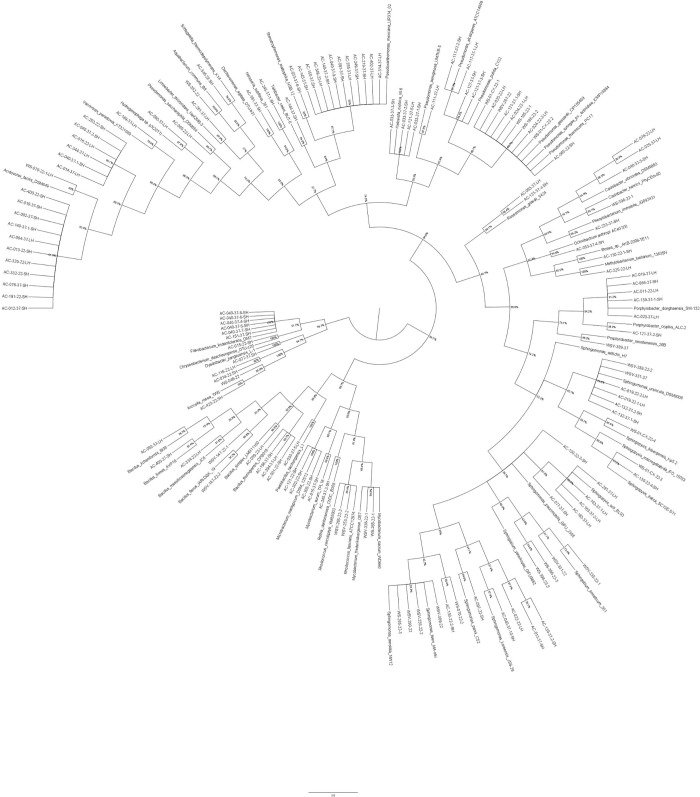
Phylogenetic tree of aircraft water isolates. The analysis involved 196 nucleotide sequences. Cluster analysis was based upon the neighbour-joining method. Numbers at branch points are percentages of 1000 bootstrap re samplings that support the topology of the tree.

The organisms identified were mostly Gram-negative, which is corroborative of previous studies of water systems. A phylogenetic tree of the identified isolates, which was generated using MEGA 6.1, can be seen in Fig 2. Two classes of Gram-positive bacteria, Actinobacteria and Bacilli, were sparsely represented however, making up 7% of the overall classes identified (Fig 1). The predominance of β-Proteobacteria (32%) and γ-Proteobacteria (30%), closely followed by α-Proteobacteria (25%) in aircraft drinking water is similar to other studies of static drinking water systems e.g. water treatment plants in Paris, USA and South China [5, 12, 33], and of bottled mineral water with similar genera and different species identified [34]. The emphasis in this study was to assess if the ecological diversity varied between the source water, WSV and the aircraft and furthermore, assess differences within the aircraft themselves. The work carried will contribute new knowledge that will be of use to industrial hygienists as well as aircraft engineers and facilitate the development of bespoke monitoring and remediation strategies for the aviation industry.

### Assessment of Bacterial Diversity across Sampling Locations

To assess variation in ecological diversity across sampling location (water supply, WSV and long and short- haul aircraft), a Pearson’s chi square assessment was undertaken and a statistical difference was found to be evident in bacterial diversity (X^2^ = 39.220, p = 0.009). This suggests that the ecology of aircraft water is variable and resultantly, poses issues for potential monitoring and/or remediation. Specifically, the presence of Flavobacteria is notably absent in both the WSV and the source water, but is present in both aircraft types (long and short haul). The identified Flavobacterium belonged to the genus *Chryseobacterium* with *C*. *indologenes* found to be present in both aircraft. A recent study has demonstrated that C. *indologenes* is capable of biofilm formation [35] and thus this may be indicative of biofilm presence within the on-board water distribution system. Furthermore, this species has previously been shown to survive in chlorinated waters, and has even been recovered from water systems within hospitals [36]. Evidence for biofilm formation within on-board water distribution systems is substantiated further by the increased bacterial loads present on-board aircraft relative to both the source and WSV water [25]; biofilms attached to the surface of a 100 mm diameter pipe have been shown to contain 25 times more bacterial cells per unit length than the adjacent bulk water [37]. The results suggest that chlorination alone will not ensure sufficient water quality when potential biofilm formation occurs within a water distribution system. Consequently, incorporation of an additional treatment system within the on-board water distribution system may be necessary to ensure a safe water supply. Alternatively, systematic removal of biofilm build up through flushing should be assimilated into the aircraft maintenance schedule.

### Assessment of Bacterial Diversity across Aircraft (Long and Short Haul)

To further elucidate the ecological diversity of bacteria across aircraft, variance across specifically the long and short haul aircraft was assessed. Differences in bacterial class diversity were apparent (Fig 1) but not necessarily obvious between the groups; larger numbers of β-Proteobacteria were classified within short haul aircraft and a notable absence of Actinobacteria classified within long haul aircraft. To quantifiably assess the differences across groups, a Pearson’s Chi Square was conducted to compare the effect of aircraft haul on the distribution of bacterial class. A significant difference across (X^2^ = 15.475, p = 0.030) the aircraft was observed, suggesting that the distribution systems across the aircraft harbour differences in bacterial diversity.

The reason for this variation is unknown. However, bacteria of the species Mycobacteria were found to be present only in the short haul distribution system and research has suggested that this species will thrive through biofilm formation, but only if it is not outcompeted by other species. In particular, the dominance of γ-Proteobacteria in the short haul distribution system may outcompete the Actinobacteria class bacteria, due to their rapid growth rate [38]. The γ-Proteobacteria identified are predominantly of the *Pseudomonas* genus, known for efficient and rapid biofilm formation [39] and thus the need for a biofilm assessment and remediation is corroborated further.

### Assessment of Bacterial Diversity within Aircraft (Fwd and Aft Galley)

**Fig 3:**
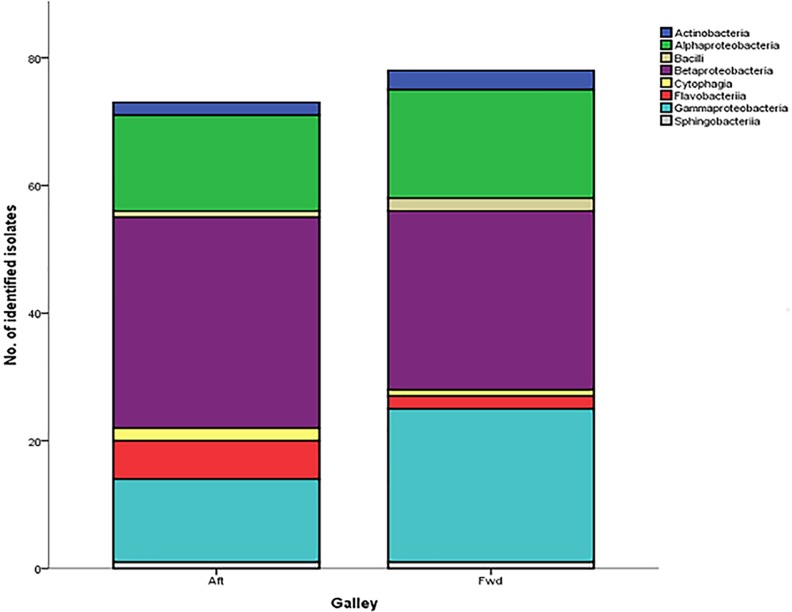
Number of isolates within each bacterial class identified across the aft and fwd galley of a fleet of short haul aircraft.

Within aircraft, water is distributed between two galleys, the forward (fwd) and the aft galley. On the short haul aircraft, water is supplied to both galleys from one water tank located in the middle of the aircraft, but through separate water lines. On the long haul aircraft there are two water tanks located towards the tail of the plane but with separate water pipes; water is drawn from both tanks at the same time. As such, an assessment of differentiation in bacterial ecology was undertaken to assess if variations to the distribution systems affects diversity. In the case of short-haul aircraft (Fig 3), there was no discernible or significant (X^2^ = 6.513, p = 0.481) difference in bacterial diversity across the galleys and all identified bacterial classes are represented regardless of galley position.

**Fig 4:**
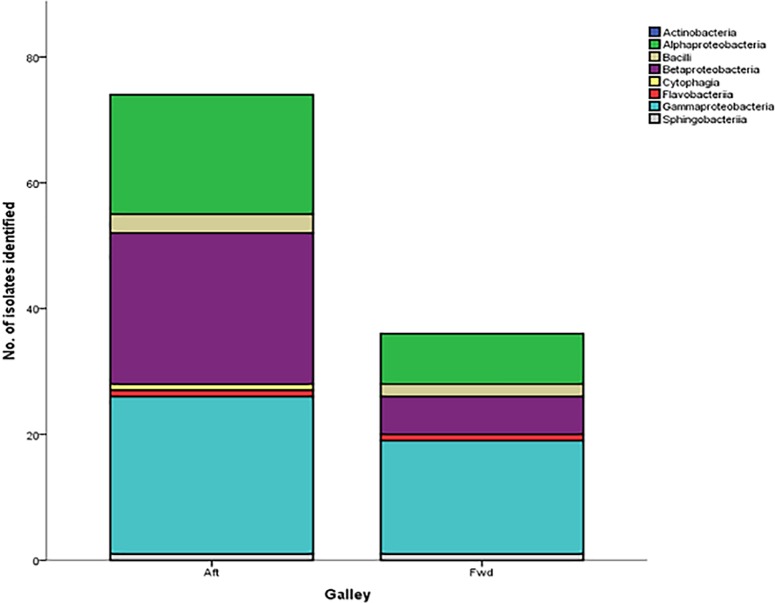
Number of isolates within each bacterial class identified across the aft and fwd galley of a fleet of long haul aircraft.

For long haul aircraft, a difference in bacterial diversity, particularly the bacterial load of β-Proteobacteria identified in the aft galley and the absence of Cytophagia bacteria in the fwd galley was noted (Fig 4), although no significant difference was present (X^2^ = 5.103, p = 0.531).

### Potential Risks

A significant diverse range of identified bacterial isolates (308) were recovered from Water Source, WSV, and from water taps in the galleys of both short- and long-haul designated aircraft. In the lifetime of the study coliforms were only detected once, initially at low levels, from a water sample of a long-haul aircraft returning to the base airport and subsequently isolated and identified as *Escherichia coli*. Notification of a city water mains break [40] at the departing airport initiated a quarantine of the on-board water and a complete draining and sanitising of the aircraft water system upon landing. Subsequent sampling and analysis were negative for *E*. *coli*.

While no water-borne pathogenic bacteria such as enterotoxigenic *E*. *coli*, *Legionella*, *Enterococcus* and *Clostridia*, etc. were found many of the bacteria identified are potentially pathogenic. These bacteria can survive the chlorination regime possibly through the formation of biofilm. *Burkholderia pseudomallei* were isolated from a WSV. This is of great concern as *B*. *pseudomallei* is a highly pathogenic bacterium that causes the disease melioidosis. While the disease is usually communicated via skin contact cases where the source was contaminated drinking water have also been found [41]. Opportunistic pathogens capable of causing infections in vulnerable individuals were also found such as the *Burkholderia cepacia* complex (BCC), *Pseudomonas aeruginosa*, *Stenotrophomonas maltophilia*, *Acinetobacter baumannii*, *Ralstonia pickettii*, *Pseudomonas fluorescens* and *Sphingomonas paucimobilis* [42–48]. BCC strains can infect and cause lung decline in patients with cystic fibrosis [49]. These bacteria could potentially act as a reservoir for the spread of antibiotic resistance. These results demonstrate the importance of vigilance, monitoring, reporting and analysis of drinking water for aircraft.

## Conclusion

To the best of our knowledge this is the first comprehensive study undertaken on microbial diversity in aircraft drinking water. Bacterial classes identified in this study are typical; representing the bacterial diversity of drinking water found in other studies, notwithstanding that drinking water is collected on occasions from multiple water sources and airports in different countries. It is acknowledged that the bacterial isolates found in this study may cause illness especially in sections of the population with underlying diseases such as immunocompromised individuals. It is important to note that no official reports of illness resulting from the consumption of aircraft drinking water could be identified in literature. However, enforcement of regulations and continued watchfulness by national health authorities, voluntary international structures such as the International Air Transport Association Drinking-Water Quality Pool (IDQP) and airlines themselves, is needed to keep the risk of contactable diseases from aircraft water at bay.

## Supporting Information

S1 Table Sequence Identification Information. (XLSX)

S2 Table bioMérieux API 20 NE Identification Information. (XLSX)

S3 Table Vitek Identification Information. (XLSX)

## References

[1] Guillot E, Loret JF, Coalition GWR. Waterborne Pathogens: Review for the Drinking-water Industry: IWA Publishing; 2010.

[2] Palumbo F, Ziglio G, Van der Beken A. Detection methods for algae, protozoa and helminths in fresh and drinking water. Padstow Cornwall UK: Wiley and Sons Ltd; 2008.

[3] Williams MM, Domingo JWS, Meckes MC, Kelty CA, Rochon HS. Phylogenetic diversity of drinking water bacteria in a distribution system simulator. J. Appl. Microbiol. 2004;96(5):954–64. 10.1111/j.1365-2672.2004.02229.x 15078511

[4] Eichler S, Christen R, Höltje C, Westphal P, Bötel J, Brettar I, et al Composition and dynamics of bacterial communities of a drinking water supply system as assessed by RNA- and DNA-based 16S rRNA gene fingerprinting. Appl. Environ. Microbiol. 2006;72(3):1858–72. 10.1128/AEM.72.3.1858-1872.2006 16517632 PMC1393179

[5] Poitelon J-B, Joyeux M, Welté B, Duguet J-P, Prestel E, Lespinet O, et al Assessment of phylogenetic diversity of bacterial microflora in drinking water using serial analysis of ribosomal sequence tags. Water Res. 2009;43(17):4197–206. 10.1016/j.watres.2009.07.020 19665751

[6] Harrigan WF. Laboratory methods in food microbiology London: Academic Press; 1998 p. 298–305.

[7] Zhang Y, He Q. Characterization of bacterial diversity in drinking water by pyrosequencing. Wa. Sci. Tech. 2013;13(2):358–67.

[8] Fenchel T. Bacterial Ecology. eLS (Online Library): John Wiley & Sons, Ltd; 2001.

[9] Ridgway HF, Olson BH. Chlorine resistance patterns of bacteria from two drinking water distribution systems. Appl. Environ. Microbiol. 1982;44(4):972–87. 7149722 10.1128/aem.44.4.972-987.1982 PMC242125

[10] Szewzyk U, Szewzyk R, Manz W, Schleifer K-H. Microbiological safety of drinking water. Annu. Rev. Microbiol. 2000;54(1):81–127. 11018125 10.1146/annurev.micro.54.1.81

[11] Poitelon J-B, Joyeux M, Welté B, Duguet J-P, Prestel E, DuBow M. Variations of bacterial 16S rDNA phylotypes prior to and after chlorination for drinking water production from two surface water treatment plants. J. Ind. Microbiol. Biotechnol. 2010;37(2):117–28. 10.1007/s10295-009-0653-5 19908076

[12] McCoy S, VanBriesen J. Temporal variability of bacterial diversity in a chlorinated drinking water distribution system. J. Environ. Eng. 2012;138(7):786–95.

[13] LeChevallier MW, Babcock TM, Lee RG. Examination and characterization of distribution system biofilms. Appl. Environ. Microbiol. 1987;53(12):2714–24. 3435140 10.1128/aem.53.12.2714-2724.1987 PMC204187

[14] Daly B, Betts WB, Brown AP, O'Neill JG. Bacterial loss from biofilms exposed to free chlorine. Microbios. 1998;96(383):7–21. 10347898

[15] Council Directive (EC) 98/83/EC. of 3 November 1998 on the quality of water intended for human consumption. OJ, 5.12.98, L330:32–54

[16] Regulation (EC) No 178/2002/EC. of 28 January 2002 of the European Parliament and of the Council laying down the general principles and requirements of food law, establishing the European Food Safety Authority and laying down procedures in matters of food safety. OJ, 1/2/2002; L31:31–23. Brussels.

[17] US EPA. Understanding the Safe Drinking Water Act. 2004, [online].

[18] US EPA. National Primary Drinking Water Regulations: Drinking Water Regulations for Aircraft Public Water Systems. 40 CFR Part 141. EPA–HQ–OW–2005–0025; FRL–8967–9. Agency UEP, US Environmental Protection Agency, Washington DC, 2009.

[19] World Health Organisation (WHO). Mode of Travel: Health Consideration, International Travel and Health. World Health Organisation, Geneva Pp14–31, 2011

[20] Bartram J., Water safety plan manual: step-by-step risk management for drinking-water suppliers 2009: World Health Organization.

[21] Mouchtouri VA, Bartlett CLR, Diskin A, Hadjichristodoulou C. Water Safety Plan on cruise ships: A promising tool to prevent waterborne diseases. Sci. Total. Environ. 2012;429:199–205. 10.1016/j.scitotenv.2012.04.018 22608187

[22] Public Health Laboratory Service and the Association of Port Health Authorities, The Microbiological Quality of Water Onboard Aircraft. 2003.

[23] Harbison, I. (2013) Airlines: Keep it clean [online], Aircraft Cabin Mangement, available: http://www.aircraftcabinmanagement.com/feature/clean-water-on-board [accessed 15 Oct 2015].

[24] Mangili A, Gendreau MA. Transmission of infectious diseases during commercial air travel. Lancet. 2005;365(9463):989–96. 10.1016/S0140-6736(05)71089-8 15767002 PMC7134995

[25] Handschuh H, O'Dwyer J, Adley CC. Bacteria that travel: The quality of aircraft water. Int. J. Environ. Res. Publ. Health. 2015;12(11):13938–55. 10.3390/ijerph121113938 PMC4661625 26529000

[26] BS ISO 5667–21:2010. Water Quality-Sampling-Part 21: Guidance on sampling of drinking water distributed by tankers or means other than distribution pipes. British Standards Institution, London, 2010.

[27] BS ISO 5667–5:2006 Water Quality-Sampling-Part 5: Guidance on sampling of drinking water from treatment works and piped distribution systems. British Standards Institution, London, 2006.

[28] Altschul SF, Gish W, Miller W, Myers EW, Lipman DJ. Basic local alignment search tool. J. Mol. Biol. 1990;215(3):403–10. 10.1016/S0022-2836(05)80360-2 2231712

[29] Thompson JD, Higgins DG, Gibson TJ. CLUSTAL W: improving the sensitivity of progressive multiple sequence alignment through sequence weighting, position-specific gap penalties and weight matrix choice. Nucleic. Acids. Res. 1994;22(22):4673–80. 7984417 10.1093/nar/22.22.4673 PMC308517

[30] Saitou N, Nei M. The neighbor-joining method: a new method for reconstructing phylogenetic trees. Mol. Biol. Evol. 1987;4(4):406–25. 3447015 10.1093/oxfordjournals.molbev.a040454

[31] Ryan MP, Adley CC, Pembroke JT. The use of MEGA as an educational tool for examining the phylogeny of antibiotic resistance genes *In*: Méndez-Vilas A, editor. Microbial Pathogens and Strategies for Combating Them: Science, Technology and Education. 1 Badajoz, Spain: Formatex; 2013 p. 736–43.

[32] Tamura K, Stecher G, Peterson D, Filipski A, Kumar S. MEGA6: Molecular evolutionary genetics analysis Version 6.0. Mol. Biol. Evol. 2013; 30(12):2725–9. 10.1093/molbev/mst197 24132122 PMC3840312

[33] Lu P, Chen C, Wang Q, Wang Z, Zhang X, Xie S. Phylogenetic diversity of microbial communities in real drinking water distribution systems. Biotechnol. Bioprocess Eng. 2013;18(1):119–24.

[34] Falcone-Dias MF, Centrón D, Pavan F, Candido da Silva M, Naveca FG, Victor Costa de S, et al Opportunistic Pathogens and Elements of the Resistome that Are Common in Bottled Mineral Water Support the Need for Continuous Surveillance. PLoS One. 2015;10(3):e0121284 10.1371/journal.pone.0121284 25803794 PMC4372423

[35] Lo HH, Chang SM. Identification, characterization, and biofilm formation of clinical *Chryseobacterium gleum* isolates. Diagn. Microbiol. Infect. Dis. 2014;79(3):298–302. 10.1016/j.diagmicrobio.2014.01.027 24796989

[36] Calderón G, García E, Rojas P, García E, Rosso M, Losada A. *Chryseobacterium indologenes* infection in a new-born: a case report. J. Med. Case. Rep. 2011;5(1):10. 21235776 10.1186/1752-1947-5-10 PMC3025965

[37] Berry D, Xi C, Raskin L. Microbial ecology of drinking water distribution systems. Curr. Opin. Biotechnol. 2006;17(3):297–302 10.1016/j.copbio.2006.05.007 16701992

[38] Pinhassi J, Berman T. Differential Growth Response of Colony-Forming α- and γ-Proteobacteria in Dilution Culture and Nutrient Addition Experiments from Lake Kinneret (Israel), the Eastern Mediterranean Sea, and the Gulf of Eilat. Appl. Environ. Microbiol. 2003;69(1):199–211. 10.1128/AEM.69.1.199-211.2003 12513996 PMC152472

[39] Coughlan A, Ryan MP, Cummins NM, Towler MR. The response of *Pseudomonas aeruginosa* biofilm to the presence of a glass polyalkenoate cement formulated from a silver containing glass. J. Mater. Sci. 2011;46(1):285–7.

[40] Levenson M, Daley B. A ‘catastrophic' rupture hits region's water system. The Boston Globe. 2010 May 2 [online], available: http://www.boston.com/news/local/massachusetts/articles/2010/05/02/a_catastrophic_rupture_hits_regions_water_system/?page=2 [Accessed 08 November 2016].

[41] Limmathurotsakul D, Wongsuvan G, Aanensen D, Ngamwilai S, Saiprom N, Rongkard P, et al Melioidosis caused by *Burkholderia pseudomallei* in drinking water, Thailand, 2012. Emerg. Infect. Dis. 2014;20(2):265–8. 10.3201/eid2002.121891 24447771 PMC3901481

[42] de Bentzmann S, Plésiat P. The *Pseudomonas aeruginosa* opportunistic pathogen and human infections. Environ. Microbiol. 2011;13(7):1655–65. 10.1111/j.1462-2920.2011.02469.x 21450006

[43] Brooke JS. *Stenotrophomonas maltophilia*: an Emerging Global Opportunistic Pathogen. Clin. Microbiol. Rev. 2012;25(1):2–41. 10.1128/CMR.00019-11 22232370 PMC3255966

[44] Peleg AY, Seifert H, Paterson DL. *Acinetobacter baumannii*: Emergence of a Successful Pathogen. Clin. Microbiol. Rev. 2008;21(3):538–82. 10.1128/CMR.00058-07 18625687 PMC2493088

[45] Ryan MP, Pembroke JT, Adley CC. *Ralstonia pickettii*: a persistent Gram-negative nosocomial infectious organism. J. Hosp. Infect. 2006;62(3):278–84. 10.1016/j.jhin.2005.08.015 16337309

[46] Ryan MP, Adley CC. *Ralstonia* spp.: emerging global opportunistic pathogens. Eur. J. Clin. Microbiol. Infect. Dis. 2014;33(3):291–304. 24057141 10.1007/s10096-013-1975-9

[47] Scales BS, Dickson RP, LiPuma JJ, Huffnagle GB. Microbiology, Genomics, and Clinical Significance of the *Pseudomonas fluorescens* Species Complex, an Unappreciated Colonizer of Humans. Clin. Microbiol. Rev. 2014;27(4):927–48. 10.1128/CMR.00044-14 25278578 PMC4187640

[48] Ryan MP, Adley CC. *Sphingomonas paucimobilis*: a persistent Gram-negative nosocomial infectious organism. J. Hosp. Infect. 2010;75(3):153–7. 10.1016/j.jhin.2010.03.007 20434794

[49] Folescu TW, da Costa CH, Cohen RWF, Neto OCdC, Albano RM, Marques EA. *Burkholderia cepacia* complex: clinical course in cystic fibrosis patients. BMC Pulm. Med. 2015;15:158 10.1186/s12890-015-0148-2 26642758 PMC4672471

